# Low Latency Global Carbon Budget Reveals Late 2024 Carbon Losses and Contrasting Early 2025 Land Sink Recovery Signals

**DOI:** 10.1002/advs.77369

**Published:** 2026-09-01

**Authors:** Piyu Ke, Philippe Ciais, Yitong Yao, Stephen Sitch, Wei Li, Xiaomeng Du, Xiaofan Gui, Ben Poulter, Thomas Colligan, Auke M. van der Woude, Joram Hooghiem, Wouter Peters, Zhu Liu, Zhu Deng, Zhe Jin, Xiangjun Tian, Yilong Wang, Junjie Liu, Sudhanshu Pandey, Chris O'Dell, Jiang Bian, John Miller, Xin Lan, Jefferson Goncalves De Souza, Michael O'Sullivan, Pierre Friedlingstein, Julien Alléon, Yi Xi, Daniel S. Goll, Lei Zhu, Guido R. van der Werf, Shilong Piao, Frédéric Chevallier

**Affiliations:** ^1^ Laboratoire des Sciences du Climat et de l'Environnement LSCE/IPSL, CEA‐CNRS‐UVSQ, Université Paris‐Saclay Gif‐sur‐Yvette France; ^2^ Department of Earth and Environmental Engineering Columbia University New York USA; ^3^ Faculty of Environment, Science and Economy University of Exeter Exeter UK; ^4^ Department of Earth System Science Ministry of Education Key Laboratory for Earth System Modeling Institute for Global Change Studies Tsinghua University Beijing China; ^5^ Machine learning group Microsoft Research Beijing China; ^6^ Spark Climate Solutions Covina California USA; ^7^ Environmental Sciences Group Department of Meteorology and Air Quality Wageningen University Wageningen The Netherlands; ^8^ Department of Geography The University of Hong Kong Hong Kong SAR China; ^9^ Institute of Carbon Neutrality Sino‐French Institute for Earth System Science College of Urban and Environmental Sciences Peking University Beijing China; ^10^ State Key Laboratory of Tibetan Plateau Earth System Environment and Resources (TPESER) Institute of Tibetan Plateau Research Chinese Academy of Sciences Beijing China; ^11^ Jet Propulsion Laboratory California Institute of Technology Pasadena California USA; ^12^ Cooperative Institute for Research in the Atmosphere Colorado State University Fort Collins Colorado USA; ^13^ National Oceanic and Atmospheric Administration Global Monitoring Laboratory Boulder Colorado USA; ^14^ Cooperative Institute for Research in Environmental Sciences University of Colorado Boulder Boulder Colorado USA; ^15^ Laboratoire de Météorologie Dynamique IPSL CNRS ENS Université PSL Sorbonne Université, École Polytechnique Paris France

**Keywords:** global carbon budget, land carbon sink, El Niño, atmospheric CO_2_ growth rate, low latency carbon monitoring

## Abstract

The 2023/24 El Niño strongly reduced land carbon uptake, but the persistence of this anomaly after surface cooling remains uncertain. Here we quantify the July 2024–June 2025 global CO_2_ budget using low‐latency fossil emission estimates, three DGVMs, machine learning ocean flux emulators and OCO‐2‐constrained atmospheric inversions. The atmospheric CO_2_ growth rate was 2.62 ± 0.08 ppm yr^−1^, 6.5% above the 2013–2022 July‐to‐June mean. DGVMs estimate that the net land sink was 1.32 ± 0.19 GtC yr^−1^ weaker than the 2015–2022 July‐to‐June mean, whereas combining bottom‐up and top‐down constraints gives a smaller deficit of 0.49 GtC yr^−1^. The annual anomaly is dominated by late‐2024 land carbon losses. Early‐2025 recovery, however, is method‐dependent: DGVMs retain a weak annual land‐sink deficit, while all inversions indicate fluxes close to the reference mean and a stronger‐than‐normal northern sink in late spring 2025. Ocean uptake shows no global weakening. A statistical decomposition links global land flux variability mainly to temperature, with terrestrial water storage contributing more strongly at regional scales. These results identify Northern Hemisphere land‐sink recovery as a central uncertainty in low‐latency carbon‐budget assessments.

## Graphical abstract

Low latency carbon budget estimates for July 2024–June 2025 combine atmospheric CO_2_ growth rates, fossil emissions, ocean uptake, DGVM land fluxes, and OCO‐2 inversions. The budget shows that late‐2024 land carbon losses dominate the annual anomaly, while early‐2025 recovery differs between bottom‐up models and top‐down inversions, especially in Northern Hemisphere extratropics.

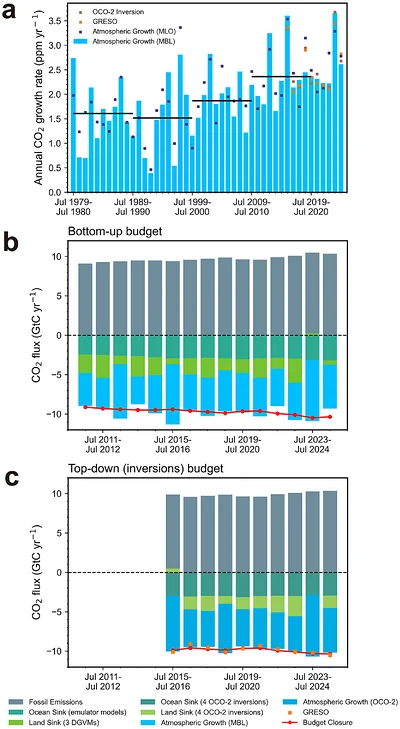

## 1 Introduction

By mid‐2025, the global air temperature had cooled by 0.26°C below the exceptional warming anomaly of 2023/24, but had not returned to normal conditions. January–June 2025 was still the second‐warmest first half‐year in NOAA's 176‐year record [1], and the 12‐month period from July 2024 to June 2025 remained 1.55°C above the 1850–1900 pre‐industrial level [2]. Over the tropical Pacific, weak La Niña conditions prevailed in early 2025, and then transitioned to ENSO‐neutral conditions by May–June 2025 [3, 4]. Regional hydroclimate anomalies persisted after the end of the 2023/24 El Niño, with severe drought during the Amazon dry season in late 2024 associated with exceptional fire emissions, and drier‐than‐average conditions in parts of South America and Africa into early 2025 [5, 6, 7]. Our previous analysis and the Global Carbon Budget 2025 (GCB2025) showed a strong reduction of land carbon uptake in the year 2024 [5]. Here, we analyze how the July 2024–June 2025 annual land carbon anomaly was partitioned between late‐2024 deficits and early‐2025 fluxes, and which regions contributed most to the source and sink anomalies.

**FIGURE 1:**
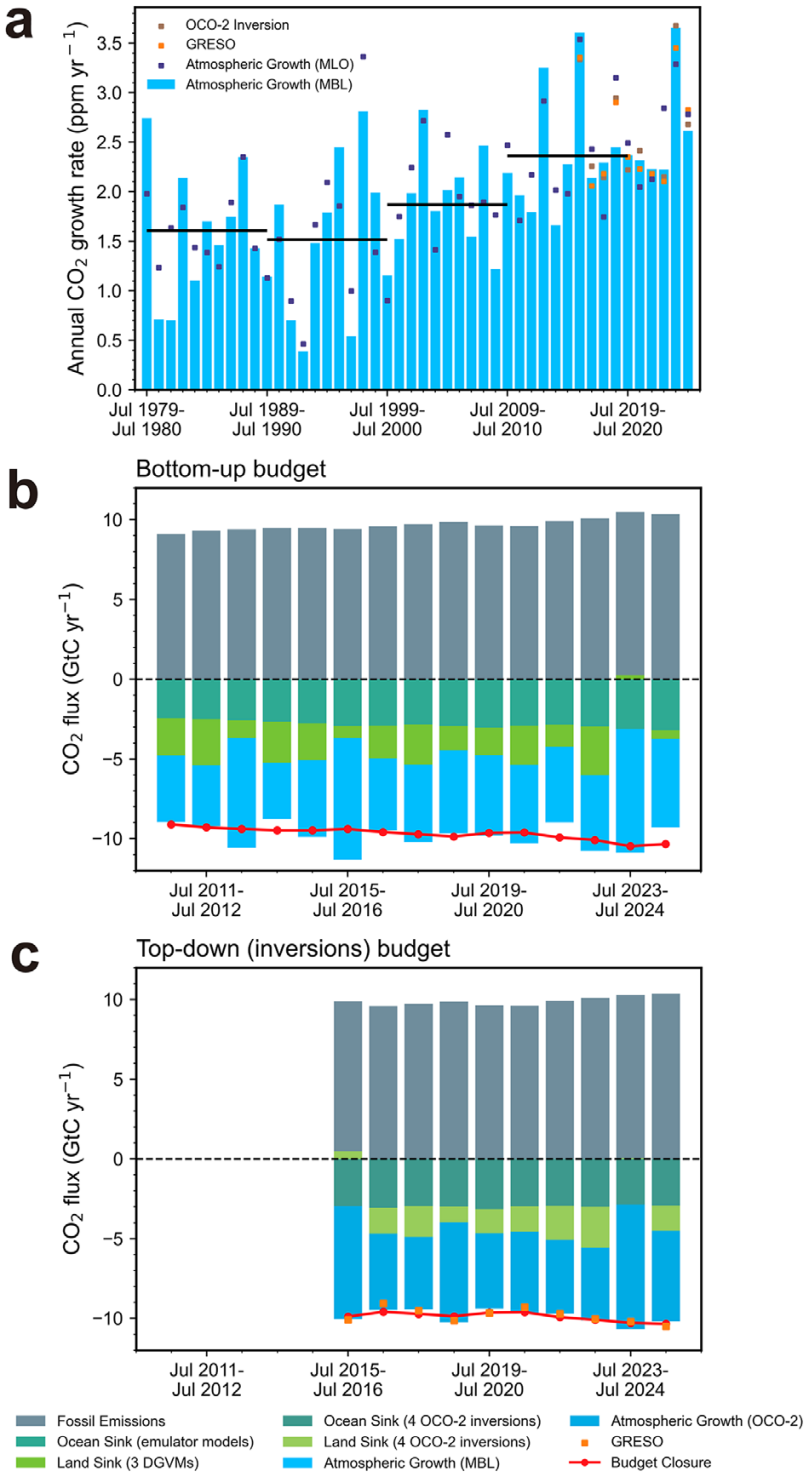
Atmospheric CO_2_ growth rate (1979–2025) and carbon budget (2010–2025), calculated for July–June annual periods. (a) Annual June‐July to June‐July growth rate from marine boundary layer surface stations (MBL, blue bars), the Mauna Loa station (MLO, dark blue squares), OCO‐2 (dark brown squares), and the Growth Rates Using Satellite Observations approach from ref. [11] (GRESO, orange squares). MBL is the average of many surface locations, which gives a better estimate of the global increase in atmospheric CO_2_ compared to the single high‐altitude MLO estimate. (b) Global CO_2_ bottom‐up budget obtained with our estimates of fossil CO_2_ emissions, DGVM‐based net land CO_2_ flux, ocean sink from ocean model emulators, and annual CO_2_ growth rates from MBL. The red curve is −1 * fossil emissions, and the difference between the bars and this curve is the imbalance of the bottom‐up budget. (c) Global CO_2_ top‐down budget obtained with our estimates of fossil CO_2_ emissions and inversions assimilating OCO‐2 atmospheric CO_2_ mixing ratios. Panel (b) uses the NOAA MBL atmospheric growth rate as the atmospheric accumulation constraint for the bottom‐up budget. In panel (c), the atmospheric accumulation term is based on the OCO‐2‐constrained growth rate, consistent with the use of OCO‐2 observations in the inversion ensemble. The orange squares show the independent GRESO growth‐rate estimate derived directly from OCO‐2 retrievals. The sensitivity of the bottom‐up budget residual to using NOAA MBL, OCO‐2, or GRESO growth rates is reported in Table S1.

The atmospheric CO_2_ growth rate provides an integrated constraint for assessing whether global carbon sinks had fully recovered by mid‐2025. Using the global average CO_2_ mixing ratio measurements of marine boundary layer sites (MBL) from the National Oceanic and Atmospheric Administration (NOAA) network [8, 9], we estimate an atmospheric CO_2_ growth rate of 2.62 ± 0.08 ppm yr^−1^ from July 2024 to June 2025, lower than the 3.66 ± 0.11 ppm yr^−1^ recorded during the El NIño from July 2023 to June 2024, but still above the 2013–2022 July‐to‐June mean of 2.46 ± 0.01 ppm yr^−1^ (0.56 ppm yr^−1^ 1‐std year‐to‐year variability) (Figure 1a). An independent growth rate estimate derived from OCO‐2 satellite observations [10] using the Growth Rates Using Satellite Observations (GRESO) framework [11] gives a value of 2.83 ± 0.10 ppm yr^−1^ comparable to NOAA, while the OCO‐2‐based estimate gives a growth rate of 2.68 ± 0.10 ppm yr^−1^ (Figure 1a). The agreement between the NOAA MBL record and satellite‐based estimates provides a robust basis for attributing the roles of land and ocean CO_2_ fluxes during the post‐El Niño period.

We update the global and regional CO_2_ budget for the period from July 2024 to June 2025 using a previously described framework [5, 12] that combines top‐down and bottom‐up constraints. The main questions addressed here are how much of the July 2024–June 2025 annual anomaly reflected late‐2024 carbon losses?; whether land fluxes in early 2025 had returned to the 2015–2022 July‐to‐June mean?; how the annual anomaly was partitioned between the tropics and the Northern Hemisphere; and to what extent drought‐related carbon losses persisted in South America and Africa into 2025. To address these questions, we combine low‐latency estimates of fossil fuel and cement CO_2_ emissions [13, 14, 15], three dynamic global vegetation models (DGVMs) [16, 17, 18, 19, 20, 21], machine‐learning emulators of monthly net CO_2_ fluxes trained on the ocean and land models of the Global Carbon Budget 2025 [15, 22], and four atmospheric inversions constrained by OCO‐2 observations [23, 24, 25, 26, 27]. To better represent fire‐related carbon losses, we corrected DGVM prognostic and unconstrained fire emissions using the observation‐based Global Fire Emissions Database (GFED4.1s) [28] and the Global Fire Assimilation System (GFAS, https://atmosphere.copernicus.eu/global‐fire‐monitoring) (see Methods). Our inversion ensemble is particularly valuable to assess tropical flux changes because OCO‐2 provides denser sampling over the tropics than the surface network, yet with many clouds and limited data during wet seasons.

## 2 Results and Discussion

### 2.1 The Global Carbon Budget From July 2024 to June 2025 Shows a Partial Recovery of the Land Sink

Figure 1 shows that the decline in atmospheric CO_2_ growth rate from the extreme value of July 2023 to June 2024 was accompanied by a recovery of the global sink, yet it remained below the 2015–2022 July‐to‐June mean. Global fossil fuel and cement CO_2_ emissions during July 2024 to June 2025 were 10.35 GtC yr^−1^ with a range of 10.33–10.37 GtC yr^−1^ from two estimates [13, 14, 15]. This range reflects the spread between the two estimates rather than a formal uncertainty estimate. In the bottom‐up budget (Figure 1b), the three DGVMs estimated a net land sink of 0.55 ± 0.31 GtC yr^−1^, with interannual anomalies from the low‐latency simulations and the mean flux level calibrated to TRENDY S3 (see Methods). The ocean models’ emulators have an ocean sink of 3.19 ± 0.65 GtC yr^−1^. The DGVM emulator ensemble estimated a terrestrial sink (without land‐use change) of 3.27 ± 1.16 GtC yr^−1^. Neither the three low‐latency DGVM estimates nor the 20‐DGVM emulator estimates include the Replaced Sinks and Sources (RSS) post‐processing correction introduced in GCB2025. These DGVM‐based estimates are therefore reported on a pre‐RSS basis and are not directly equivalent to RSS‐corrected GCB2025 S_LAND_ estimates (see Methods). Combined with the NOAA MBL atmospheric CO_2_ growth rate, these bottom‐up terms imply a budget residual of 1.05 ± 0.70 GtC yr^−1^ (Monte Carlo 95% confidence interval: −0.44 GtC yr^−1^ to 2.23 GtC yr^−1^), that is, an additional sink required to close the bottom‐up budget. The propagated uncertainty was estimated by sampling the atmospheric CO_2_ growth rate, ocean‐product members, and DGVM land‐flux members (see Methods and Table S1). Because the 95% confidence interval includes zero, this residual is not significantly different from zero at the 95% confidence level. Sensitivity to the atmospheric growth‐rate product is substantial: the residual decreases to 0.92 ± 0.71 GtC yr^−1^ using the OCO‐2 growth rate and to 0.60 ± 0.71 GtC yr^−1^ using GRESO, with corresponding land sinks required to close the budget of 1.47 ± 0.67 and 1.15 ± 0.67 GtC yr^−1^, compared with 1.61 ± 0.65 GtC yr^−1^ under NOAA MBL (Table S1). The 1.05 ± 0.70 GtC yr^−1^ term therefore represents a bottom‐up budget imbalance under the NOAA MBL growth‐rate constraint, rather than an independently constrained flux component.

In the top‐down budget (Figure 1c) we use an OCO‐2 inversion from Carbon Tracker Europe [27] in addition to the three OCO‐2 inversions of previous assessments, GONGGA, CAMS and CMS‐Flux. The four OCO‐2 inversions estimate a net land sink of 1.58 ± 0.73 GtC yr^−^ **^1^** and an ocean sink of 2.93 ± 0.17 GtC yr^−1^. The inversion land sink was about 0.16 GtC yr^−^ **^1^** higher than the mean July‐to‐June strength during 2015–2022. The inversion budget has no imbalance and closes the budget to within 0.15 GtC yr^−1^ (Figure 1c) for July 2024‐June 2025 when using the atmospheric growth rate from OCO‐2.

Averaging the bottom‐up and top‐down estimates gives a global net land sink of 1.06 ± 0.40 GtC yr^−1^ and an ocean sink of 3.06 ± 0.34 GtC yr^−1^ for July 2024 to June 2025. Relative to the El Niño weak land sink or small land source from July 2023 to June 2024, the global land sink strengthened by 1.21 GtC yr^−1^, but remained 0.49 GtC yr^−1^ weaker than the 2015–2022 July‐to‐June mean. The ocean term did not indicate a weakening of global ocean uptake: the combined ocean sink was 0.10 ± 0.34 GtC yr^−1^ higher than the reference mean, and a separate low‐latency ocean‐product diagnostic showed a modest positive July–June global ocean‐sink anomaly, with small integrated tropical Pacific anomalies in early 2025 despite regional spatial structure (Figure S10).

The land response is central to explaining why the atmospheric CO_2_ growth rate remained above its average value despite a cooling of 0.26°C after the late‐2024 peak (Figure S2). Because fossil emissions changed only modestly and our ocean model emulators do not point to an ocean‐driven weakening of global uptake, the remaining budget tension primarily concerns land fluxes and atmospheric‐growth‐rate constraints. The negative July 2024–June 2025 land‐sink anomaly relative to the 2015–2022 July‐to‐June mean, is mainly explained by deficits in late 2024 rather than by a continuing deficit during January–June 2025. Yet, in early 2025, the DGVMs indicate a remaining annual land sink deficit relative to the 2015–2022 mean, whereas the inversions indicate that land fluxes had returned close to the reference mean. Overall, the July 2024–June 2025 land‐sink anomaly was dominated by late‐2024 deficits, with the extent of recovery during early 2025 differing strongly between DGVMs and inversions.

### 2.2 Late‐2024 Deficits Dominated the Annual Land Flux Anomaly Despite Contrasting Recovery in Early 2025

The negative annual land sink anomaly between July 2024 and June 2025 was not uniform throughout the year. It was dominated by weak sink conditions in late 2024 [5], whereas the first half of 2025 was characterized by a return toward the long‐term mean, whose magnitude differed between DGVMs and inversions. Relative to the reference July‐June mean of 2015–2022, the land flux showed an anomalous source of 1.13 ± 0.41 GtC in July–December 2024 in the DGVMs and of 0.41 ± 0.40 GtC in the inversions. In January–June 2025, the DGVMs still showed a weak source anomaly of 0.19 ± 0.29 GtC, whereas the inversions showed a positive sink anomaly of 0.57 ± 0.47 GtC. The second half of the year 2024 therefore accounted for 86% of the annual July 2024–June 2025 land‐sink anomaly in the DGVMs, the remaining 14% reflecting an incomplete recovery during the first half of 2025. In contrast, the inversions give a more rapid rebound, where the sink anomaly in the first half of 2025 effectively offset the abnormal carbon losses during the second half of 2024.

**FIGURE 2:**
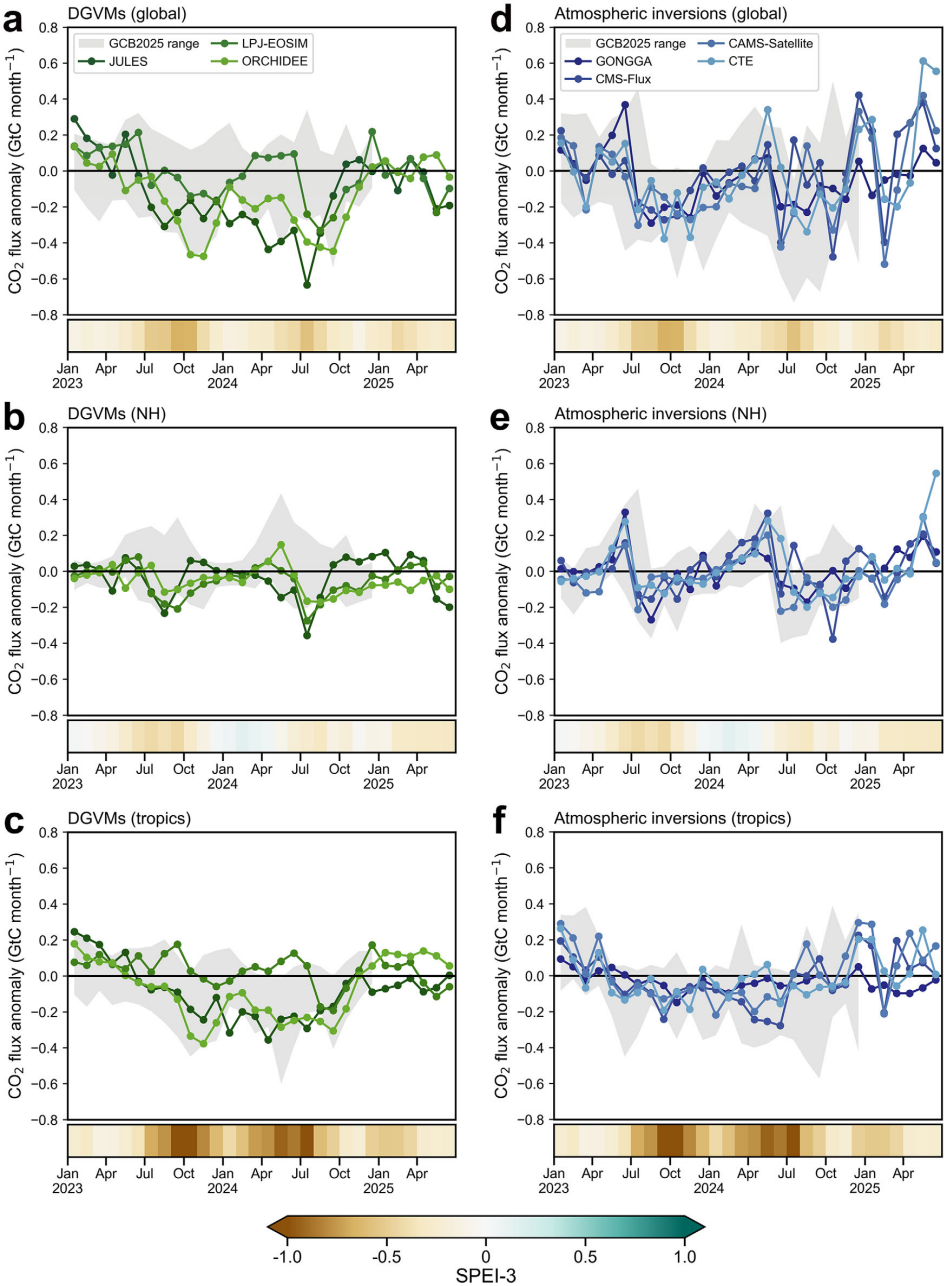
Evolution of monthly net land CO_2_ flux anomalies in three latitude domains. Anomalies from the three DGVMs used in this study from January 2023 to June 2025 for global land (a), Northern Hemisphere non‐tropical land (NH) (b), and tropical land (c). Same for the four atmospheric inversions: global land (d), Northern Hemisphere non‐tropical land (e), and tropical land (f). Anomalies are calculated relative to the 2015–2022 monthly mean in each domain. Positive values indicate anomalously strong land CO_2_ uptake, whereas negative values indicate reduced uptake or anomalous carbon release. The grey envelope denotes the inter‐model spread of the broader GCB2025 land‐model ensemble, providing context for the broader structural spread of land‐model estimates beyond that sampled by the three low‐latency DGVMs. The colored strips below each panel show the domain‐mean 3‐month Standardized Precipitation‐Evapotranspiration Index (SPEI‐3) from the ERA5‐Drought dataset. Negative SPEI‐3 values indicate drier‐than‐normal conditions, and positive values indicate wetter‐than‐normal conditions [52].

The monthly evolution of land‐flux anomalies provides further insight (Figure 2a,d). In the DGVMs, monthly negative land flux anomalies (source anomalies) persisted from July 2024 to November 2024, reaching a peak source anomaly of 0.42 ± 0.20 GtC month^−1^ in July 2024 (Figure 2a); the inversions showed a comparable period of source anomaly from July 2024 to November 2024, yet with a smaller peak source anomaly of 0.28 ± 0.16 GtC month^−1^ in October 2024 compared with the DGVMs (Figure 2d). In both DGVMs and inversions, land uptake therefore remained anomalously weak through late 2024, consistent with drought‐ and fire‐related carbon losses during the 2024 South American dry season. Source anomalies then alleviated from January 2025 onward during boreal winter and spring 2025, with a quasi‐neutral land flux anomaly of 0.03 ± 0.05 GtC month^−1^ in the DGVMs and a weak sink anomaly of 0.09 ± 0.08 GtC month^−1^ in the inversions over January–June 2025. Nevertheless, 3 out of 6 months in the DGVMs and 2 out of 6 months in the inversions still showed source anomalies over January–June 2025, indicating that the recovery remained partial and temporally intermittent.

### 2.3 Tropical and Extratropical Land Flux Anomalies Jointly Shaped the Weak Annual Sink From July 2024 to June 2025

The annual land flux anomaly from July 2024 to June 2025 differed between latitude bands (Figure 2). In the DGVMs, tropical lands contributed a source anomaly of 0.48 ± 0.47 GtC, accounting for 36.7% of the global land source anomaly, while Northern Hemisphere extratropical lands contributed a large source anomaly of 0.79 ± 0.43 GtC (60.2%) and Southern Hemisphere extratropical lands had a negligible contribution of 0.04 ± 0.03 GtC (3.1%). In the inversions, by contrast, tropical lands showed a sink anomaly of 0.36 ± 0.69 GtC, while Northern Hemisphere extratropical lands were nearly neutral with a flux anomaly of 0.04 ± 0.38 GtC and Southern Hemisphere extratropical lands were a source anomaly of 0.18 ± 0.34 GtC. The annual land flux anomaly therefore reflected contrasting latitudinal differences in the two approaches: the DGVMs predict both tropical and Northern Hemisphere extratropical reductions in uptake, whereas the inversions predict a tropical sink‐anomaly and a near‐neutral annual Northern Hemisphere extratropical anomaly. To assess whether this contrast was driven by individual ensemble members, we added a member‐level diagnostic across the main latitude bands and tropical regions (Figure S8). All three DGVMs simulated a global land source anomaly for July 2024–June 2025, with member values ranging from 1.10 to 1.44 GtC yr^−1^ and an ensemble mean of 1.32 ± 0.19 GtC yr^−1^. Inversions spanned from a source anomaly of 0.75 GtC yr^−1^ to a sink anomaly of 0.89 GtC yr^−1^, with an ensemble‐mean weak sink anomaly of 0.16 ± 0.68 GtC yr^−1^. Thus, the annual DGVM–inversion contrast is not only a difference between ensemble means, but a difference between the sampled bottom‐up and top‐down ranges.

The monthly net flux anomalies of DGVMs and inversions are displayed in Figure 2b,c,e,f. During July–December 2024, the DGVM source anomaly was shared almost equally between the tropics (54%) and Northern Hemisphere extratropics (53.5%). The most negative tropical anomaly reached 0.22 ± 0.07 GtC month^−1^ in September 2024 (Figure 2c). In the inversions, by contrast, the late‐2024 source anomalies were concentrated in the Northern extratropics where hot conditions persisted (Figure 2e) while the tropics showed a weak sink anomaly (Figure 2f). The peak monthly tropical source anomaly in the inversions was much smaller than in the DGVMs, reaching 0.07 GtC month^−1^ in October 2024. During January–June 2025, tropical anomalies weakened and shifted to quasi‐neutral values of 0.02 ± 0.08 GtC month^−1^ in the DGVMs and 0.04 ± 0.08 GtC month^−1^ in the inversions. A complementary comparison is provided by machine‐learning emulators trained on the 20 monthly‐output DGVMs of GCB2025 (Figure S7). Although these emulators are not used in the main bottom‐up budget, they reproduce the same broad tropical temporal structure as the three low‐latency DGVMs: a shift to source anomalies in late 2024 followed by much weaker anomalies in early 2025, albeit with smaller amplitude. This suggests that the timing of the tropical recovery is not unique to the three low‐latency DGVMs, even if the magnitude of the anomaly remains model‐dependent. During January–June 2025, Northern extratropical lands had a near‐neutral source anomaly of 0.03 ± 0.03 GtC month^−1^ in the DGVMs, and a weak mean sink anomaly of 0.07 ± 0.06 GtC month^−1^ in the inversions. The 20‐DGVM emulator ensemble provides a broader statistical estimate for land‐model behavior and structural spread, while the three directly updated DGVMs provide the low‐latency bottom‐up budget term used for budget closure.

The member‐level diagnostic refines this interpretation by showing that the tropical DGVM–inversion difference is mainly a late‐2024 difference. During July–December 2024, all DGVMs simulated tropical source anomalies larger than those inferred by the inversions, with a DGVM source anomaly range of 0.33–1.81 GtC yr^−1^, whereas inversions ranged from a source anomaly of 0.25 GtC yr^−1^ to a sink anomaly of 1.06 GtC yr^−1^. Tropical South America accounted for much of this difference between approaches. In this region, the DGVM source anomaly range during July–December 2024 was 0.51‐1.63 GtC yr^−1^, compared with an inversion range from a source anomaly of 0.37 GtC yr^−1^ to a weak sink anomaly of 0.03 GtC yr^−1^. By January–June 2025, however, the tropical DGVM and inversion ranges overlapped, indicating that the tropical discrepancy was associated primarily with the magnitude of late‐2024 carbon losses rather than with a persistent tropical disagreement throughout early 2025.

A different seasonal flux change is found in the Northern Hemisphere extratropics. The DGVM and inversion ranges overlap during July–December 2024, but differ during January–June 2025 and differ more during AMJ 2025. In AMJ 2025, all inversion members indicate sink anomalies, while all DGVM members indicate source anomalies. The DGVM ensemble gives a source anomaly of 0.71 ± 0.44 GtC yr^−1^, whereas the inversion ensemble gives a sink anomaly of 1.99 ± 0.90 GtC yr^−1^. This corresponds to a cumulative DGVM–inversion difference of 0.67 GtC over AMJ 2025. Leave‐one‐member‐out tests show that this difference remains after removing any single DGVM or inversion member, indicating that the AMJ 2025 Northern Hemisphere extratropical discrepancy is not caused by a single ensemble outlier. This DGVM‐inversion difference is not spatially universal: Tropical Africa and Southeast Asia show overlapping DGVM and inversion ranges for all periods examined. The largest DGVM‐inversion differences are therefore concentrated in two contexts: late‐2024 tropical South America and early‐2025 Northern Hemisphere extratropical fluxes.

### 2.4 South American and African Droughts Prolonged Regional Abnormal Carbon Losses Into 2025

**FIGURE 3:**
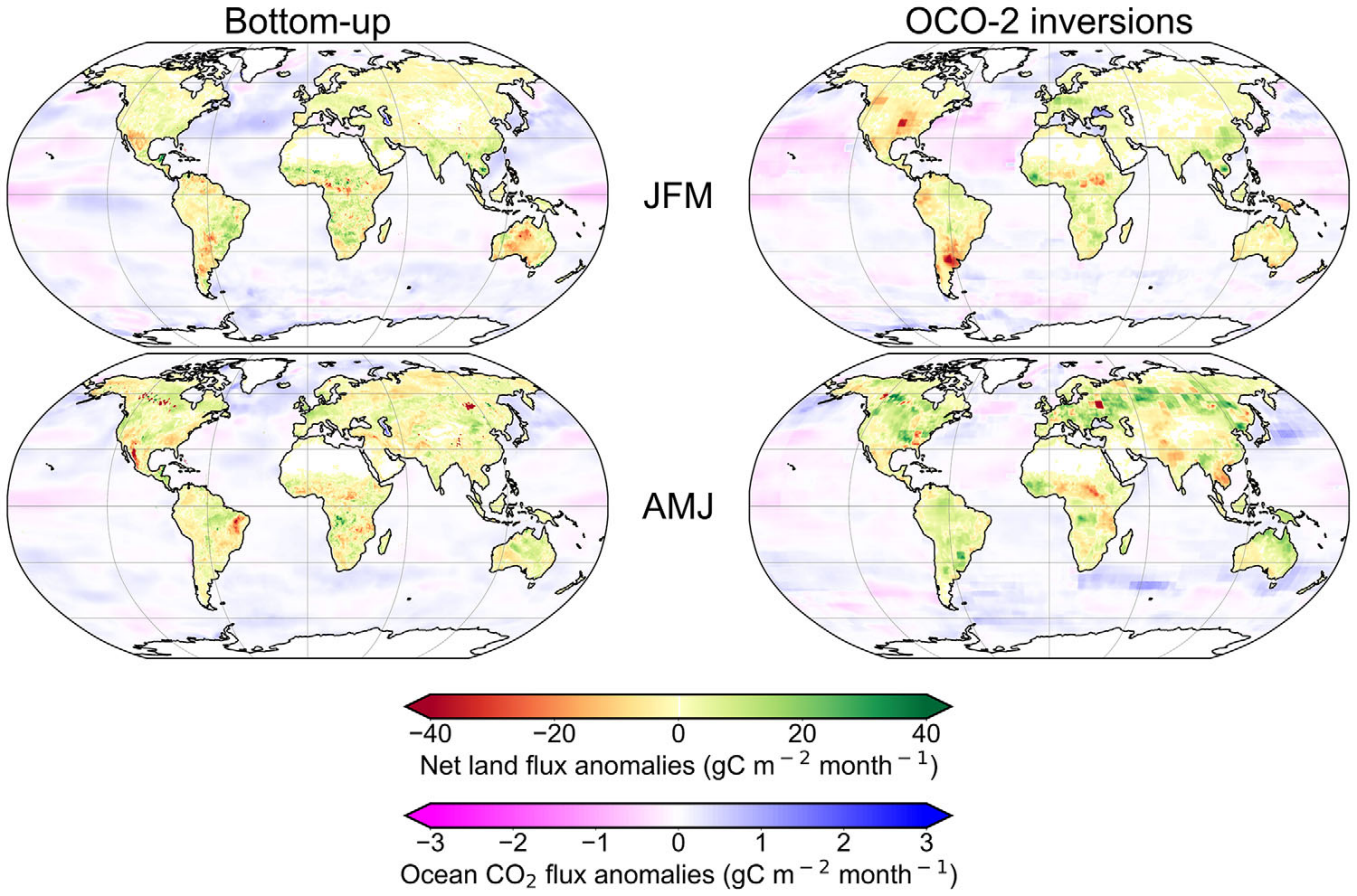
Net land and ocean CO_2_ flux anomalies for January to March (JFM) and April to June (AMJ) 2025. Anomalies are calculated relative to the 2015–2022 average of the DGVM models (left column, mean of three models) and the OCO‐2 inversions (right column, mean of four models). Positive values represent anomalous CO_2_ uptake from the atmosphere by the land (green) or ocean (blue).

**FIGURE 4:**
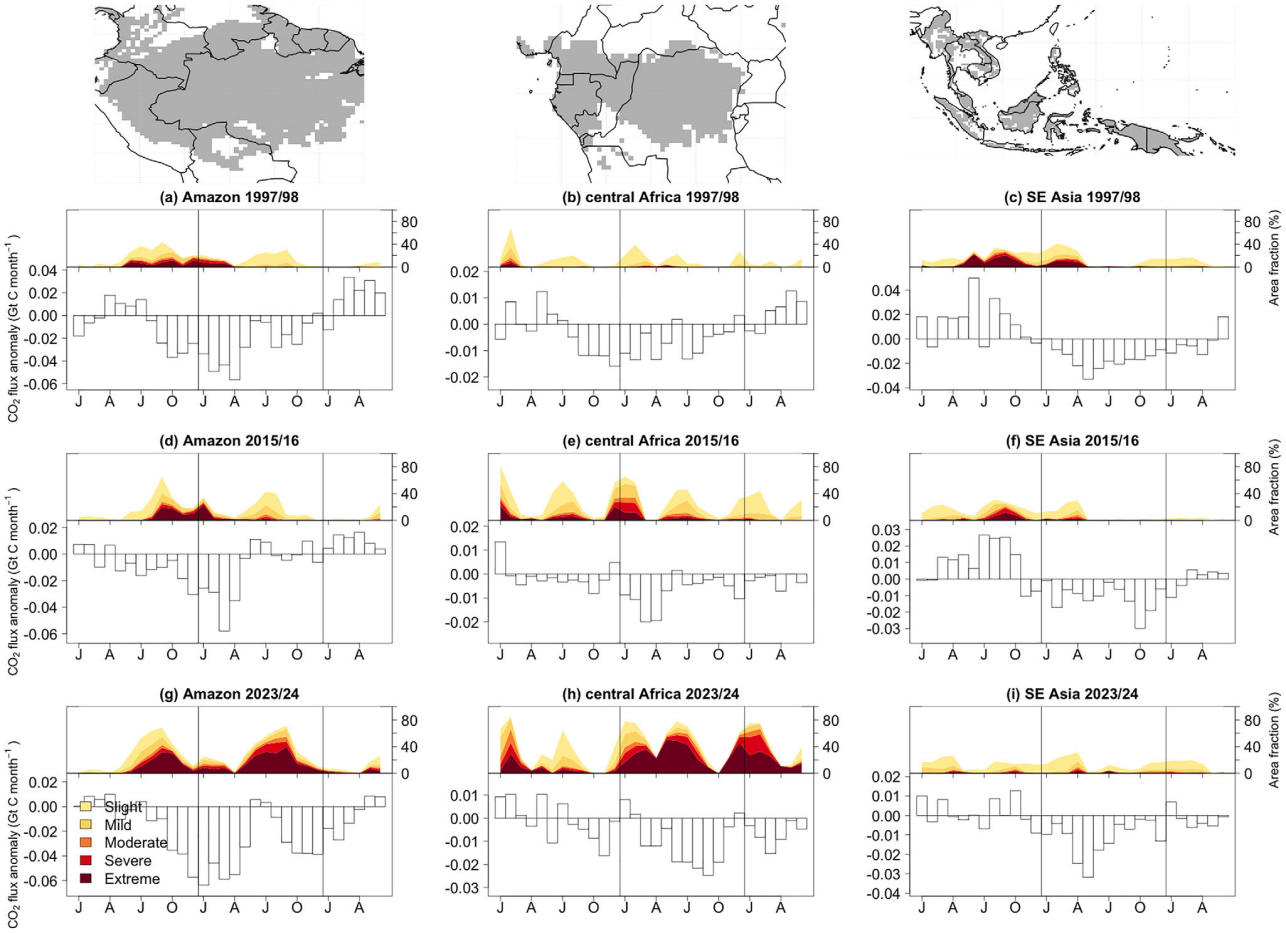
Drought timing and extent of the three El Niño events with net CO_2_ flux anomalies. The upper part of each subplot displays the fraction of each tropical continental area in grey under drought for different intensities. Fractional area under each drought severity category is calculated for different severities based on Z‐score‐transformed monthly cumulative water deficits. The lower part displays monthly net land CO_2_ flux anomalies from the mean of three DGVMs (ORCHIDEE, LPJ‐EOSIM and JULES) calculated relative to the 5‐year pre‐event baseline for each El Niño event: 1992–1996 for 1997/98, 2010–2014 for 2015/16, and 2018–2022 for 2023/24. (a–c) For the 1997/98 El Niño in the Amazon, Central Africa, and tropical Southeast Asia. (d–f) Same for the 2015/16 El Niño. (g–i) Same for the 2023/24 El Niño. The anomalies for this El Niño are only simulated until June 2025.

The spatial patterns of the flux anomalies shown in Figure 3 indicate that South America was the region with the largest land source anomaly in 2025, while Africa exhibited weaker and more spatially heterogeneous anomalies. Because some local hotspots in Figure 3 differ between DGVMs and inversions, we added DGVM‐minus‐inversion difference maps as a diagnostic check of spatial disagreement (Figure S9). These maps identify where the two approaches diverge, but the regional interpretation below is based primarily on region‐integrated anomalies and on the temporal evolution of drought, rather than on individual grid‐cell extrema. The continental contrasts are consistent with the temporal evolution of drought in the major tropical forest regions (Figure 4). Tropical South America experienced the strongest and most persistent drought signal during the 2023/24 El Niño and again during the 2024 dry season, and its land‐flux anomalies remained sources well into early 2025. Central Africa showed a weaker but longer‐lived hydroclimatic legacy, with drought conditions and source flux anomalies persisting after the end of El Niño yet with a smaller magnitude than in South America. Tropical Southeast Asia, by contrast, exhibited a short drought‐induced source anomaly and a faster return toward neutral or sink‐like flux anomalies.

Over South America in January–March 2025, both bottom‐up models and OCO‐2 inversions indicated persistent source anomalies, with regionally integrated values of 0.04 ± 0.08 GtC in the DGVMs and 0.16 ± 0.12 GtC in the inversions (Figure 3). Yet spatial distributions within South America differ between both approaches: the DGVMs place the source anomalies across tropical and subtropical South America, whereas the inversions emphasize localized source anomalies in the southern and southeastern parts of the continent. By April–June 2025, the DGVMs show a close‐to‐neutral South American source anomaly of 0.03 ± 0.17 GtC and inversions show a sink anomaly of 0.17 ± 0.16 GtC. Thus, South America remained the clearest tropical regional source anomaly in early 2025 mainly during the first quarter, while the AMJ signal became weaker and more method‐dependent. Extending the findings of our previous low‐latency assessment for 2024, these results show that the South American drought legacy persisted into early 2025, but weakened by AMJ as the regional anomaly approached neutral values in DGVMs and shifted to a sink anomaly in inversions.

Over Africa in January–March 2025, there is a more heterogeneous situation. In the DGVMs, continent‐wide flux anomalies were small, shifting from a weak sink anomaly of 0.09 ± 0.03 GtC in January–March 2025 to a weak source anomaly of 0.03 ± 0.10 GtC in April–June 2025, indicating near‐compensation between source and sink regions. In the inversions, Africa showed weak sink anomalies in both January–March (0.06 ± 0.23 GtC) and April–June (0.01 ± 0.25 GtC) as seen in Figure 3. Inversions show spatially coherent source anomalies over southern Africa and Sudan and sink anomalies in the Congo basin (Figure 3). African land flux anomalies therefore did not simply vanish after the 2024 drought ended.

Over Southeast Asia in January–March 2025, neutral anomalies of 0.02 ± 0.04 GtC in the DGVMs and 0.01 ± 0.11 GtC in the inversions prevailed. In April–June 2025, neutral anomalies of 0.00 ± 0.02 GtC in the DGVMs and 0.01 ± 0.04 GtC in the inversions continued. At the annual July 2024–June 2025 scale, Southeast Asia had a sink anomaly of 0.06 ± 0.07 GtC in the DGVMs and 0.15 ± 0.12 GtC in the inversions, confirming that it did not contribute substantially to the weaker global land sink.

The data in Figure 4 show the evolution of monthly flux anomalies from the DGVMs across the Amazon, Central African, and South East Asian forests together with the fraction of forest area under different drought‐severity classes diagnosed from precipitation‐based cumulative water deficit (CWD) [29] from 2023 to June 2025. This period is compared with previous El Niño events in 1997/98 and 2015/16. Clearly, the fraction of forest area under extreme and severe drought was much larger in the most recent period in the Amazon and Central Africa but not in South East Asia. Severe drought persisted in the Amazon after the El Niño in the second half of 2024, and prevailed in central Africa from January to September 2024, and from November 2024 to June 2025, thus for a longer period than in the Amazon. Severe drought was much weaker and more short‐lived in Southeast Asia. The DGVM flux anomalies showed persistent negative anomalies over much of 2023–2025, and were stronger than during the two previous El Niño periods. In particular, DGVMs simulate two peaks of strong source anomalies in the Amazon from October 2023 to May 2024, and from August 2024 to February 2025. DGVMs also simulate an extended source anomaly from March 2024 to October 2024 in Central Africa followed by a smaller source anomaly centered on April 2025. In South East Asia, despite no severe drought anomaly, a modest source anomaly persisted through much of 2024, with the strongest negative anomalies in boreal spring to autumn 2024.

### 2.5 Temperature and Water‐Storage Contributions

**FIGURE 5:**
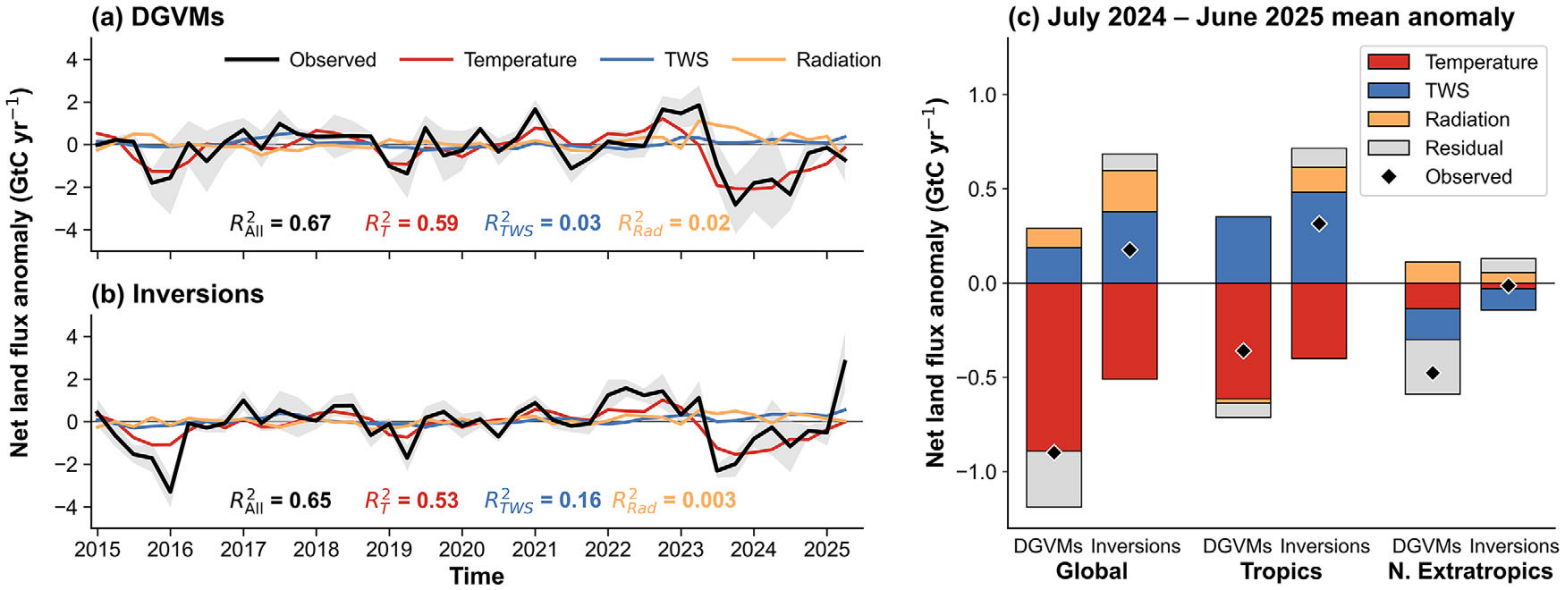
Climate contributions to the weak global land carbon sink during July 2024–June 2025. (a, b) Global net land CO_2_ flux anomalies aggregated to non‐overlapping 3‐month means from 2015 to June 2025 for the 3 low‐latency DGVMs (a) and the atmospheric inversion ensemble (b). The black line shows the ensemble‐mean observed anomaly, and the grey shading denotes ±1 s.d. across ensemble members. Red, blue, and orange lines show the contributions of temperature (T), terrestrial water storage (TWS), and radiation, respectively, estimated from a pixel‐wise linear climate model fitted at 2° × 2° resolution using non‐overlapping 3‐month anomalies. (c) Mean net land CO_2_ flux anomaly for July 2024–June 2025, decomposed into contributions from temperature, TWS, radiation, and a residual term for global land, the tropics (23.5°S–23.5°N), and the Northern Hemisphere extratropics (>23.5°N), shown separately for the DGVM and inversion ensembles. Black diamonds indicate the total observed anomaly, equal to the sum of the stacked components. All anomalies are expressed relative to the 2015–2022 mean. Negative values indicate reduced net land CO_2_ uptake, or anomalous carbon release, relative to the reference period.

ENSO‐driven annual fluctuations of tropical temperature (T) and total water storage (TWS) were found to explain most of the inter‐annual fluctuations of global land fluxes [30, 31]. Warmer conditions generally reduce net land CO_2_ uptake, whereas wetter conditions tend to enhance it. Statistical models using temperature, water availability, and radiation (RAD) were also shown to explain variations of local fluxes [32], with regional compensations of water availability anomalies letting T emerge as the main driver of global land flux anomalies. In this study, CWD and TWS are used as complementary moisture diagnostics rather than interchangeable water‐stress metrics. CWD characterizes the timing and severity of tropical‐forest droughts in Figure 4, following previous studies of Amazon and tropical‐forest drought impacts based on cumulative water deficit or related precipitation‐deficit metrics [29, 33, 34], whereas TWS provides a globally consistent observational constraint on terrestrial water‐storage anomalies for the statistical attribution in Figure 5 and has been linked to atmospheric CO_2_ growth rate variability [30, 31]. To quantify these contributions, we decomposed DGVM and inversion land‐flux anomalies using a pixel‐wise linear regression model fitted at 2° × 2° resolution to non‐overlapping 3‐month anomalies. Over the 2015–2022 training period, the full T–TWS–RAD reconstruction explained 65% of the DGVM variance and 68% of the inversion variance at the global scale. For the independent 2023–June 2025 period, the corresponding out‐of‐sample R^2^ values were 0.51 and 0.47, respectively. Skill was higher in the tropics, with out‐of‐sample R^2^ values of 0.71 for DGVMs and 0.85 for inversions, but the Northern Hemisphere extratropics showed no positive out‐of‐sample skill (R^2^ = ‐0.27 and −0.06, respectively), indicating that the linear model did not capture the amplitude of northern anomalies during the 2023–2025 extremes. As a complementary descriptive metric, the squared correlations shown in Figure 5a,b indicates that the temperature contribution had the strongest full‐period co‐variation with observed global anomalies (R2 = 0.62 for DGVMs and 0.53 for inversions), whereas TWS (0.08 and 0.16) and RAD (0.03 and 0.003) were smaller at the global scale. This contrast between the emergent global role of temperature and the stronger regional roles of TWS and RAD is consistent with the scale dependence reported by Ref. [32].

The decomposition of the July 2024–June 2025 mean anomaly clarifies how T and TWS controls differed between DGVMs and inversions in Figure 5c (see also Figures S3 and S6). In the DGVMs, the global mean anomaly was a source anomaly of 1.32 GtC yr^−1^. Warmer temperature provided the largest source contribution (1.01 GtC yr^−1^), while TWS and RAD provided smaller and compensating sink contributions of 0.13 and 0.15 GtC yr^−1^, respectively. The remaining unexplained term was a source residual of 0.59 GtC yr^−1^, indicating that our linear regression models did not fully capture the magnitude of the DGVM anomaly. In the inversions, by contrast, the global mean anomaly was a weak sink anomaly of 0.18 GtC yr^−1^ despite a temperature‐driven source contribution of 0.51 GtC yr^−1^, because TWS and RAD provided sink contributions of 0.38 GtC yr^−1^ and 0.22 GtC yr^−1^, leaving a residual sink anomaly of 0.09 GtC yr^−1^. TWS‐driven sink contributions, that is, wetter conditions associated with enhanced carbon uptake (Figures S3 and S6), were spatially concentrated over tropical Africa and Australia in both inversions and DGVMs, and additionally in parts of Canada in the DGVMs.

The T, TWS, and RAD contributions differed by latitude. In the tropics, the DGVM anomaly was a source anomaly of 0.49 GtC yr^−1^, driven by a temperature‐induced source contribution of 0.70 GtC yr^−1^ partly offset by a TWS sink contribution of 0.36 GtC yr^−1^; RAD was near neutral and the residual was a source term of 0.15 GtC yr^−1^. In the tropics, the inversion mean anomaly was a sink anomaly of 0.32 GtC yr^−1^, because TWS and RAD sink contributions of 0.48 GtC yr^−1^ and 0.13 GtC yr^−1^ exceeded the temperature source contribution of 0.40 GtC yr^−1^, leaving a residual sink anomaly of 0.10 GtC yr^−1^. In the Northern Hemisphere extratropics, the DGVM anomaly was a source anomaly of 0.79 GtC yr^−1^, with source contributions from temperature and TWS of 0.17 GtC yr^−1^ and 0.24 GtC yr^−1^, partly offset by a RAD sink contribution of 0.14 GtC yr^−1^, and with a large unexplained source residual of 0.52 GtC yr^−1^. The inversion anomaly in the Northern Hemisphere extratropics was close to neutral, with a weak source anomaly of 0.01 GtC yr^−1^. Because these contributions are estimated jointly in a multivariate regression, they should be interpreted as partial linear effects conditional on the other predictors, rather than as fully independent physical responses. In addition, because the pixel‐wise regressions are fitted to a limited number of non‐overlapping quarterly anomalies, we interpret the fitted coefficients only after regional aggregation and in combination with out‐of‐sample skill, quantile diagnostics, and residual terms, rather than as definitive local process sensitivities. Radiation‐related contributions should also be interpreted cautiously because ERA5 shortwave radiation may contain cloud‐ and aerosol‐related uncertainties, particularly in tropical and high‐latitude regions.

The lower skill in northern lands is central to interpreting these results. The linear reconstruction retained high rank agreement in the quantile diagnostics, but compressed anomaly amplitudes, especially in the Northern Hemisphere extratropics. Reconstructed‐versus‐observed quantile slopes were 0.40 for DGVMs and 0.26 for inversions in the Northern Hemisphere extratropics, compared with 0.78 and 0.64 in the tropics (Figure S5). This indicates that the linear model captures the ordering of anomalies better than their amplitude in northern lands, probably because ecosystem lagged effects and nonlinear temperature–moisture interactions are not represented. Overall, the exceptionally high land temperature anomalies observed from June 2023 to December 2024 imposed a broad tendency toward weaker land uptake, whereas TWS anomalies acted mainly as regional compensating effects in the tropics and parts of the Southern Hemisphere, but not in northern lands, where drought‐related TWS deficits contributed to source anomalies.

These limitations are important for interpreting the contrast between DGVM and inversion recovery signals. The member‐level diagnostics show that the DGVM–inversion discrepancy is not attributable to any single ensemble member, but also not a uniform bottom‐up versus top‐down offset. The largest DGVM‐inversion differences are a late‐2024 tropical South American source anomaly amplitude difference, with larger source anomalies in DGVMs, and an AMJ 2025 Northern Hemisphere extratropical recovery‐signal difference, with inversions indicating sink anomalies while DGVMs remain source‐like. Because these contrasts occur in different regions and seasons, and because the climate‐driver model is fitted separately within each flux product, the decomposition in Figure 5 should be interpreted as a statistical attribution of anomalies within each product, not as a reconciliation of DGVM and inversion recovery signals. Plausible contributors include atmospheric transport, OCO‐2 sampling and prior‐flux structure in inversions; meteorological forcing, fire treatment, fixed land use and process sensitivities in DGVMs; and lateral carbon transport, which can decouple land‐surface carbon bookkeeping from the atmospheric signals constrained by inversions. This Northern Hemisphere discrepancy also falls within a broader, long‐standing uncertainty in the latitudinal partitioning of land CO_2_ fluxes between northern extra‐tropical and tropical lands. Previous atmospheric‐profile and inversion studies, as well as the GCB2025 synthesis, have shown that atmospheric constraints and land models can differ in the inferred strength and distribution of northern versus tropical land sinks [15, 35, 36, 37]. Recent work further suggests that disturbance, regrowth, fire treatment, and forest demography can contribute to differences between DGVM and inversion estimates of the northern land sink [38]. Our AMJ 2025 DGVM‐inversion difference should therefore be interpreted as a seasonal low‐latency expression of this broader partitioning problem, rather than as evidence that either the DGVMs or inversions alone provide a resolved process attribution. The harmonized low‐latency outputs used here do not allow these factors to be separated quantitatively, so the pace and spatial structure of early‐2025 land‐sink recovery remain a method‐dependent uncertainty.

## 3 Conclusions

The July 2024–June 2025 global carbon budget shows that the effects of the 2023/24 El Niño persisted into 2025 through a reduced terrestrial carbon sink. Although land uptake partially recovered in early 2025, atmospheric CO_2_ growth remained above average, reflecting the lingering impact of late‐2024 carbon losses. Process‐based models and atmospheric inversions agree that late‐2024 deficits shaped the annual anomaly, but differ in the amplitude and timing of recovery. The strongest diagnosed DGVM‐inversion differences occur in two specific contexts: late‐2024 tropical South America, where DGVMs simulate larger source anomalies than inversions, and AMJ 2025 Northern Hemisphere extratropics, where inversions indicate sink anomalies while DGVMs remain source‐like. These findings indicate that climate‐driven carbon cycle anomalies can outlast the underlying climate forcing, highlighting the need for low‐latency observations to track recovery and improve attribution of carbon flux variability. In particular, future studies should aim at explaining the large differences between land models and inversions by using independent products such as flux‐tower upscaled fluxes and observation‐based estimates of gross fluxes related to gross primary productivity.

## 4 Methods

### 4.1 Atmospheric CO_2_ Growth Rate (CGR)

We used the monthly time series of globally averaged marine surface (MBL) atmospheric CO_2_ dry air mole fraction covering the period from January 1979 to November 2025, and the MLO station data from March 1958 to January 2026, both provided by NOAA's Global Monitoring Laboratory (NOAA/GML) [8, 9]. The July‐June annual MBL growth rate is determined by averaging the most recent June and July months, corrected for the average seasonal cycle, and subtracting the average of the same period in the previous year (https://gml.noaa.gov/ccgg/trends/gl_gr.html). For the MLO data, the July‐June annual mean growth rate is estimated using the average of the most recent May‐August months, corrected for the average seasonal cycle, and subtracting the same four‐month average of the previous year centered on July first (https://gml.noaa.gov/ccgg/trends/gr.html). When an uncertainty is quoted for this custom July–June metric, it is estimated by simple error propagation of the monthly NOAA trend uncertainty values for the June–July endpoints, assuming independent monthly uncertainties. The reported value should therefore be interpreted as an approximate propagated 1σ uncertainty for this July–June estimate, not as NOAA's formal annual‐growth uncertainty for its standard annual‐growth product. For GRESO growth rates [11], we use OCO‐2 retrievals from both land‐ and ocean‐glint observations and aggregate them to global monthly means through a sequence of spatiotemporal averaging and filtering steps. Retrievals are first quality‐filtered (excluding soundings with XCO_2_ uncertainty > 2 ppm and “target mode” soundings) and then binned onto a 5° × 5° grid using 16‐day temporal windows. Gridded XCO_2_ values are converted to global means using area weighting, deseasonalized with a fourth‐order polynomial plus four harmonic terms, and smoothed with a 3‐point boxcar filter. We then extract June–July values and define the annual mid‐year CO_2_ concentration as the arithmetic mean of the June and July global‐average monthly CO_2_ values. Differences between OCO‐2 and NOAA MBL growth rates are also expected, as NOAA MBL data may not fully represent whole‐atmosphere growth rates due to the limited sampling of surface flask measurements [39].

### 4.2 Global Fire CO_2_ Emissions

Both the Global Fire Emissions Database version 4.1 including small fire burned area (GFED4.1s) [28], and the Global Fire Assimilation System (GFAS, https://atmosphere.copernicus.eu/global‐fire‐monitoring) from Copernicus Atmosphere Monitoring Service (CAMS) are used to derive monthly global fire CO_2_ emissions. GFED4.1s combines satellite information on fire activity and vegetation productivity to estimate the gridded monthly burned area and fire emissions, and has a spatial resolution of 0.25° × 0.25° [28]. Note that GFED4.1s fire emissions for the 2017 and onward period are from the beta version. The GFAS assimilates fire radiative power (FRP) observations from satellites to produce daily estimates of biomass burning emissions. We aggregated the GFAS daily fire emissions into monthly emissions.

### 4.3 Terrestrial CO_2_ Fluxes

Terrestrial carbon fluxes are derived from the mean of three Dynamic Global Vegetation Models (DGVMs), specifically JULES, ORCHIDEE, and LPJ‐EOSIM. The methodology for estimating terrestrial carbon fluxes for the period 2010–2025 aligns with the TRENDY protocol, used in Global Carbon Budget [40], albeit with modifications due to the use of ERA5 climate forcing and the fact that land‐use forcing is not updated with such short latency. ERA5 forcing has been available since 1940 [41], but preliminary simulations with DGVMs showed some issues with precipitation forcing before 1960. The simulations performed here correspond to the S2 experiment, starting from steady‐state in 1960, with time‐varying climate and CO_2_ forcing, and land‐use fixed at 2010, as described in Ref. [42].

Since the model simulations start from a steady‐state in the 1960s, they cannot account for the carbon balance before the Industrial Revolution, which leads to an underestimation of the mean trend compared to standard DGVMs. Therefore, we calibrated them using the 2019–2022 TRENDY DGVMs simulation 3 from the Global Carbon Budget 2023 [43]. This was done by calculating, for each grid cell, the median of the carbon flux from the TRENDY models during 2019–2022 and the mean of each of the DGVMs used in this study, then subtracting from each DGVM on each grid the difference so that they match the median flux of the TRENDY models. In other words, our DGVMs are used to predict interannual anomalies, while the mean net land‐flux level is calibrated to TRENDY S3; land‐use effects represented in TRENDY S3 are therefore included in the baseline adjustment, although post‐2010 land‐use transitions are not explicitly simulated by the low‐latency S2 runs. The 2019–2022 GCB2023 TRENDY S3 baseline was retained to ensure consistency with our previous anomaly‐based low‐latency assessments. To preserve that methodological continuity, we did not apply the GCB2025 RSS post‐processing correction to the three low‐latency DGVM estimates or to the 20‐DGVM emulator estimates; both are therefore reported on a pre‐RSS basis. The baseline adjustment affects the absolute mean net land flux and budget closure, whereas the main recovery diagnostics are based on interannual anomalies and their temporal evolution relative to the 2015–2022 reference period.

ERA5 meteorological forcing data exhibit known biases in tropical precipitation during extreme climatic conditions such as the 2023/24 El Niño event [44]. Previous evaluations and comparisons with alternative precipitation products indicate that ERA5 precipitation anomalies tended to be systematically lower over critical tropical regions during the April–May 2024 drought peak. These precipitation discrepancies influenced carbon flux estimates from low‐latency DGVMs, especially through vegetation productivity and simulated fire emissions. Although replacing DGVM‐simulated fire emissions with observational fire emission data (GFED/GFAS) reduces the dependence on model‐internal fire simulations, some residual bias inevitably persists because vegetation productivity and drought‐stress responses still depend on the meteorological forcing. Currently, these uncertainties are recognized but have not yet been explicitly quantified. Systematic evaluation of the uncertainties associated with meteorological forcing datasets, along with explicit uncertainty quantification and bias corrections, will be incorporated in future updates of our low‐latency carbon budget framework.

### 4.4 Machine‐Learning Emulators of DGVMs

To provide low‐latency predictions of global land carbon fluxes for January–June 2025 (H1‐2025), we also developed deep‐learning‐based emulators of 20 Dynamic Global Vegetation Models (DGVMs) participating in the Global Carbon Budget 2025 [15]. Each emulator was trained on monthly data from 2000 to 2024 at a 0.5°×0.5° spatial resolution. Input features included atmospheric CO_2_ mole fraction, vegetation maps, lagged carbon fluxes from the previous 12 months, and eight meteorological variables from ERA5 [41]: surface pressure, surface downward short‐wave radiation, surface downward long‐wave radiation, 2‐meter temperature, total precipitation, 10‐meter wind u‐ and v‐components, specific humidity. Held‐out validation against parent DGVM outputs for 2022–2023 is summarized in Section S1. The emulators are therefore used here as a complementary low‐latency benchmark for broader land‐model behaviour and structural spread, while the three directly updated DGVMs remain the main bottom‐up budget term used for budget closure.

### 4.5 Ocean CO_2_ Fluxes

Ocean carbon fluxes are derived from a suite of emulators based on both biogeochemical models and data‐driven models. We updated estimates from 10 Global Ocean Biogeochemical Models and 9 data products included in the Global Carbon Budget 2025 to create a low‐latency framework [15, 22]. This update employs Convolutional Neural Networks (CNNs) and semi‐supervised learning techniques to capture the non‐linear relationships between model or product estimates and observed predictors. As a result, we obtain a low‐latency, monthly grid‐based dataset of global surface ocean fugacity of CO_2_ and ocean‐atmosphere CO_2_ flux data, extending to June 2025. The methodology and previous evaluation of this low‐latency ocean‐emulator framework are described in Refs. [12, 22], and the same framework was used in our previous low‐latency assessment [5]. In the present study, we use the updated product ensemble extended to June 2025 and add a period‐specific diagnostic of ocean CO_2_ flux anomalies during the 2024–2025 ENSO transition in Figure S10.

### 4.6 Anthropogenic CO_2_ Emissions

For the period from 2010 to 2024, we used global fossil fuel and cement CO_2_ emissions estimates from the latest edition of the global carbon budget [15]. For 2025, we used the average of emissions from the Carbon Monitor project based on near‐real‐time activity data from 6 sectors [13, 14] and from an updated estimate using the same methodology as the Global Carbon Budget [15], i.e., based on energy data available by the time of the publication and projections for countries with no available data. We then applied monthly emissions proportions derived from the IEA‐EDGAR monthly CO_2_ emissions dataset from the Emissions Database for Global Atmospheric Research (EDGAR) [45] to partition annual emissions into the first and second halves of each year. The same monthly distribution observed in 2024 was assumed for 2025 to estimate emissions specifically for H1‐2025.

### 4.7 Bottom‐up Budget Residual Uncertainty Propagation

We estimated the uncertainty of the bottom‐up budget residual using a Monte Carlo approach. The residual was defined as fossil CO_2_ emissions minus atmospheric accumulation, ocean uptake, and the DGVM land sink. Atmospheric accumulation was calculated from the atmospheric CO_2_ growth rate using 1 ppm CO_2_ = 2.124 GtC. For each atmospheric growth rate product, we sampled the growth rate from a Gaussian distribution using its reported mean and 1‐sigma uncertainty. We sampled ocean uptake with replacement from the 19 ocean‐product members and sampled land uptake with replacement from the three DGVM members. Fossil CO_2_ emissions were held fixed at the central low‐latency estimate because the 10.33‐10.37 GtC yr^−1^ range represents the spread between two near‐real‐time estimates rather than a formal 1‐sigma uncertainty. For each case, we generated 500,000 Monte Carlo samples and calculated the residual distribution. The uncertainty reported in Table S1 is the 1‐sigma standard deviation of this distribution, and the 95% confidence interval was calculated from the 2.fifth and 97.fifth percentiles. We assessed whether the residual differed significantly from zero by checking whether the 95% confidence interval excluded zero. The implied land sink required to close the budget was calculated as fossil CO_2_ emissions minus atmospheric accumulation and ocean uptake, with uncertainty estimated from the same atmospheric‐growth‐rate and ocean‐product sampling.

### 4.8 Atmospheric Inversions

We used four atmospheric inversions driven by the OCO‐2 satellite atmospheric CO_2_ column‐average dry air mole fraction data, which all contributed to the annual CO_2_ budget assessment. The four inversions are not fully independent in their meteorological drivers: CAMS and CTE use ERA5 meteorology, whereas GONGGA and CMS‐Flux use MERRA‐2 meteorology. Agreement among inversions should therefore be interpreted as agreement across four OCO‐2‐constrained inversion systems, but not as a fully independent sampling of atmospheric transport uncertainty.

### 4.9 CAMS Inversion

The CAMS CO_2_ atmospheric inversion (https://ads.atmosphere.copernicus.eu/datasets/cams‐global‐greenhouse‐gas‐inversion) provides global estimates of weekly greenhouse gas fluxes with a typical 4‐month latency, now at a resolution of about 90 km based on a hexagonal‐pentagonal mesh of the globe. The product used here is version FT25r2. It followed the usual production and quality control process of the CAMS products. It covers the OCO‐2 period from October 2014 to July 2025, and its mean fluxes and anomalies are close to the median of inversions used in previous assessments [43, 46]. The underlying transport model was nudged toward horizontal winds from the ERA5 reanalysis. The inferred fluxes were estimated in each horizontal grid point of the transport model with a temporal resolution of 8 days, separately for day‐time and night‐time. The prior values of the fluxes combine estimates of (i) gridded monthly fossil fuel and cement emissions (GCP‐GridFED version 2025.1 [47]) extended to year 2025 following Chevallier et al. (2020) [24] using the emission changes reported by https://carbonmonitor.org/, together with anomalies in retrievals of NO_2_ columns from the Tropospheric Monitoring Instrument (TROPOMI, offline and processing and RPRO when available, van Geffen et al., 2019 [48]), (ii) monthly ocean fluxes (Chau et al. 2024a [49], 2024b [50]), 3‐hourly (when available) or monthly biomass burning emissions (GFAS) and climatological 3‐hourly biosphere‐atmosphere fluxes taken as the 1981–2020 mean of a simulation of the ORganizing Carbon and Hydrology In Dynamic EcosystEms model, version 2.2, revision 7262 (ORCHIDEE, Krinner et al. 2005 [16]). The variational inversion accounts for spatial and temporal correlations of the prior errors, resulting in a total 1‐sigma uncertainty for the prior fluxes over a full year of 3.0 GtC∙yr^−1^ for the land pixels and of 0.2 GtC∙yr^−1^ for the marine pixels.

### 4.10 GONGGA Inversion

This system provides global estimates of CO_2_ fluxes over the period of January 2015 to June 2025 at a spatial resolution of 2° by 2.5° (latitude×longitude) with 47 layers in the vertical direction from the surface to the top of the atmosphere. It follows the production and quality control process as described in Ref. [25]. The model is driven by Modern‐Era Retrospective analysis for Research and Applications 2 (MERRA‐2) meteorological data. The 3‐h fluxes were optimized in each horizontal grid point of the transport model in each 14‐day temporal window. The prior values of the fluxes combine estimates of (i) gridded monthly fossil fuel and cement emissions (GCP‐GridFED version 2024.0 [47]) extended to year 2025 using the Global gRidded dAily CO_2_ Emission Dataset‐GRACED (https://carbonmonitor‐graced.com/) [51]; (ii) gridded monthly ocean flux from CarbonTracker 2022 *p*CO_2_‐Clim prior data, which were derived from the Takahashi et al. (2009) climatology of seawater *p*CO_2_; (iii) gridded monthly biosphere‐atmosphere CO_2_ exchange fluxes simulated by ORCHIDEE‐MICT model extended to year 2025 by repeating the fluxes in 2023; (iv) gridded monthly biomass burning emissions from the Global Fire Emissions Database (GFED; version 4.1s). The prior errors account for spatial and temporal correlations, resulting in a total 1‐sigma uncertainty for the prior global fluxes over a full year of 4.7 GtC∙yr^−1^ for land fluxes and of 0.3 GtC∙yr^−1^ for ocean fluxes.

### 4.11 CMS‐Flux Inversion

This system provides global estimates of CO_2_ fluxes over the period of Jan 2015‐ June 2025 at a spatial resolution of 4° by 5° (latitude × longitude) with 47 layers in the vertical direction from the surface to the top of the atmosphere. The inversion setups follow ref. [26]. The model is driven by Modern‐Era Retrospective analysis for Research and Applications 2 (MERRA‐2) meteorological data. CMS‐Flux optimizes ocean and land fluxes at monthly temporal resolution, assuming a known diurnal cycle from the prior land and ocean carbon fluxes. The prior fluxes include (i) gridded monthly fossil fuel and cement emissions (GCP‐GridFED version 2024.0 [47]) extended to year 2025 using the Global gRidded dAily CO_2_ Emission Dataset‐GRACED (https://carbonmonitor‐graced.com/) [51]; (ii) 3‐hourly land carbon fluxes including fire emissions from CARDAMOM; (iii) 3‐hourly ocean fluxes from either ECCO‐Darwin or daily ocean fluxes from MOM‐6. We carried out two sets of top‐down flux inversions with two different ocean prior fluxes, and the final results are the mean of the two flux inversions. CMS‐Flux assumes no spatial and temporal correlations in the prior flux uncertainties. The 1‐sigma uncertainties for land and ocean fluxes are 1.3 GtC∙yr^−1^ and 0.3 GtC∙yr^−1^ respectively.

### 4.12 CTE Inversion

The CarbonTracker Europe v3 inversion provides global estimates of CO_2_ fluxes from Jan 2015 to July 2025 using in situ observations of CO2 to constrain long‐term mean carbon fluxes, and OCO2 observations to add spatiotemporal variability on top of this. For this, we optimise the equation NEE = λ_1_ * TER_clim_ + GPP_clim_ + TER_iav_ + λ_2_ + GPP_iav_, in which *TER* and *GPP* are total ecosystem respiration and gross primary productivity from the SiB4 model. The subscripts clim and iav indicate long‐term mean and inter‐annual variability, respectively. λ_1_ is optimised using 23 years (2000‐2022) of in situ marine background observations of CO_2_, λ_2_ is subsequently optimised over 5‐day moving windows using the 2015–2025 OCO2 product. We use the transport model TM5‐MP, driven by ERA5 data.

### 4.13 Drought Characteristics

The characteristics of meteorological drought in the tropics are described by the Cumulative Water Deficit (CWD). Based on precipitation data from ERA5‐Land, we calculated monthly CWD for each pixel, as:

(1) $$\mathrm{CW}D_{m}=\min\left(0,\;\mathrm{CW}D_{m-1}+P_{m}-100\right)\;\mathrm{if}\;P_{m}<100,\;\mathrm{else}\;\mathrm{CW}D_{m}=0$$

with *m* being the month within the hydrological year spanning from October of the previous year to September of the current year. CWD_m_ is the CWD of the current month, which is equal to the CWD from the previous month (CWD_m‐1_) plus the difference between the precipitation of the current month (P_m_) and ET (assumed to be 100 mm). The water deficit accumulates over the entire hydrological year. If monthly rainfall falls below 100 mm, the forest is considered to be experiencing a water deficit. For each grid cell, the Z‐score of CWD at the i^th^ grid for the m^th^ month is calculated as:

(2) $$Z_{CWD,m.i}=\frac{CWD_{m,i}-\mu_{CWD,m,i}}{\sigma_{CWD,m,i}}\;$$

where µ_*CWD*,*m*, *i*_ and σ_*CWD*,*m*, *i*_ are the mean value and standard deviation of CWD at the i^th^ grid for the m^th^ month during the reference period. We used the monthly Z_CWD_ to identify the spatial extent, duration, severity, onset, and end of the El Niño‐related drought events in the tropical forests. Drought intensity was classified into five categories, slight (Z∈[−1,0)), mild (Z∈[−1.645, −1)), moderate (Z∈[−1.96, −1.645)), severe (Z∈[−2.576, −1.96)), and extreme (Z< −2.576).

### 4.14 Climate‐Driven Decomposition of Land‐Flux Anomalies

We decomposed land‐flux anomalies into contributions from temperature (T), terrestrial water storage (TWS), and radiation (RAD) using a pixel‐wise linear regression framework. Previous studies have shown that interannual land‐carbon variability is strongly linked to temperature and terrestrial water storage, and that radiation and water availability help explain regional flux anomalies [30, 31, 32]. Monthly anomalies of land flux, T, TWS, and RAD were calculated relative to the 2015–2022 monthly climatology at each grid cell. Land‐flux anomalies were derived from the DGVM and inversion ensembles described above, whereas T and RAD were taken from ERA5 [41] and TWS from the GRACE/FO CSR RL06 mascon product (see Data Availability). All fields were aligned to a common monthly time axis and regridded to a common 2° × 2° grid. Flux anomalies were aggregated using area‐weighted coarsening, whereas TWS and RAD were interpolated bilinearly before aggregation. Monthly anomalies were then converted to non‐overlapping 3‐month means using quarter starts in January.

At each grid cell, we fitted an ordinary least‐squares linear regression of the form

$$F^{\prime }=\beta \_T\;T^{\prime }+\beta \_W\;TWS^{\prime }+\beta \_R\;RAD^{\prime }+c$$

where primes denote non‐overlapping 3‐month anomalies, and c is an intercept. Regression coefficients were estimated over the 2015–2022 reference period and then applied to the full January 2015–June 2025 record to reconstruct the 3‐month land‐flux anomalies. Driver‐specific contributions were computed by multiplying each fitted coefficient by the corresponding predictor anomaly, and the residual term was defined as the observed anomaly minus the reconstructed anomaly.

To preserve ensemble structure, the regression was fitted separately to each DGVM and inversion member, and the resulting reconstructed time series, attribution maps, and regional contributions were then averaged across members. Global and zonal contributions shown in Figure 5c were obtained by averaging the driver‐specific fields over July 2024–June 2025 and integrating them over global land, the tropics (23.5°S–23.5°N), and the Northern Hemisphere extratropics (>23.5°N). Model performance was assessed in three complementary ways. First, reconstruction skill was quantified using R^2^ between reconstructed and observed 3‐month anomalies over the 2015–2022 training period and, independently, over January 2023–June 2025 using coefficients fitted over 2015–2022. Second, quantile–quantile diagnostics were used to evaluate whether the reconstruction captured the distribution and amplitude of anomalies for the global, tropical, and northern extratropical aggregates (Figure S5). Third, the R^2^ values annotated in Figure 5a,b were calculated as squared Pearson correlations between observed global 3‐month anomalies and the reconstructed total or individual driver contributions over January 2015–June 2025; these values summarize full‐period co‐variation rather than out‐of‐sample predictive skill.

## Author Contributions

P.C. and P.K. designed the research; P.K. and P.C. performed the analysis; P.K., P.C. and Y.Y. collected and analyzed the research data; P.C. and P.K. created the first draft of the paper; all authors contributed to the interpretation of the results and to the text.

## Conflicts of Interest

The authors declare no conflicts of interest.

## Supporting information




**Supporting File**: advs77369‐sup‐0001‐SuppMat.docx.

## Data Availability

The data from Global Carbon Budget 2025 are available at https://globalcarbonbudget.org/datahub/the‐latest‐gcb‐data‐2025/. The OCO‐2 retrievals are available at disc.gsfc.nasa.gov/datasets?page=1&keywords=OCO‐2. NOAA/GML CO_2_ data are available at https://gml.noaa.gov/ccgg/trends/. The Carbon Monitor fossil emissions dataset is available at carbonmonitor.org. The GFED 4.1s fire emissions dataset is available at geo.vu.nl/~gwerf/GFED/GFED4/. The GFAS fire emissions dataset is available at atmosphere.copernicus.eu/global‐fire‐monitoring/. The ERA5 monthly averaged data is available at cds.climate.copernicus.eu/cdsapp#!/dataset/reanalysis‐era5‐single‐levels‐monthly‐means?tab=overview. The ERA5‐Drought dataset used to derive SPEI‐3 is available from the ECMWF Cross Data Store (ECDS): Monthly drought indices from 1940 to present derived from ERA5 reanalysis (DOI: 10.24381/9bea5e16). The GRACE/FO TWS data used in this study are available at www2.csr.utexas.edu/grace/RL06_mascons.html.
