# Supplementary material for: Low Latency Global Carbon Budget Reveals Late 2024 Carbon Losses and Contrasting Early 2025 Land Sink Recovery Signals
Source: Adv Sci (Weinh). 2026 Sep 1:e77369. Online ahead of print. doi: 10.1002/advs.77369 (PMC13534783; doi:10.1002/advs.77369)
Supplement: Supplementary file 1 [file ADVS-9999-e77369-s001.docx]

Supplemental Information for

Low latency global carbon budget reveals late 2024 carbon losses and contrasting early 2025 land sink recovery signals

Piyu Ke^1,*^, Philippe Ciais^1,*^, Yitong Yao^2^, Stephen Sitch^3^, Wei Li^4^, Xiaomeng Du^4^, Xiaofan Gui^5^, Ben Poulter^6^, Thomas Colligan^6^, Auke M. van der Woude^7^, Joram Hooghiem^7^, Wouter Peters^7^, Zhu Liu^4^, Zhu Deng^8^, Zhe Jin^9^, Xiangjun Tian^10^, Yilong Wang^10^, Junjie Liu^11^, Sudhanshu Pandey^11^, Chris O’Dell^12^, Jiang Bian^5^, John Miller^13^, Xin Lan^13,14^, Jefferson Goncalves De Souza^3^, Michael O’Sullivan^3^, Pierre Friedlingstein^3,15^, Julien Alléon^1^, Yi Xi^1^, Daniel S. Goll^1^, Lei Zhu^1^, Guido R. van der Werf^7^, Shilong Piao^9^, Frédéric Chevallier^1^

1. Laboratoire des Sciences du Climat et de l'Environnement, University Paris Saclay CEA CNRS, Gif sur Yvette 91191, France
2. Department of Earth and Environmental Engineering, Columbia University, New York 10027, USA
3. Faculty of Environment, Science and Economy, University of Exeter, Exeter EX4 4QF, United Kingdom
4. Department of Earth System Science, Ministry of Education Key Laboratory

for Earth System Modeling, Institute for Global Change Studies,Tsinghua University, Beijing 100084, China

5. Machine learning group, Microsoft research, Beijing 100080, China
6. Spark Climate Solutions,Covina, CA, USA
7. Environmental Sciences Group, Dept of Meteorology and Air Quality, Wageningen University, Wageningen 6708 PB, the Netherlands
8. Department of Geography, The University of Hong Kong, Hong Kong SAR, China
9. Institute of Carbon Neutrality, Sino-French Institute for Earth System Science, College of Urban and Environmental Sciences, Peking University, Beijing 100871, China
10. State Key Laboratory of Tibetan Plateau Earth System, Environment and Resources (TPESER), Institute of Tibetan Plateau Research, Chinese Academy of Sciences, Beijing 100101, China
11. Jet Propulsion Laboratory, California Institute of Technology, Pasadena 91011, CA, USA
12. Cooperative Institute for Research in the Atmosphere, Colorado State University, Fort Collins, CO 80523, USA
13. National Oceanic and Atmospheric Administration Global Monitoring Laboratory, CO 80303, USA
14. Cooperative Institute for Research in Environmental Sciences, University of Colorado Boulder, CO 80303, USA
15. Laboratoire de Météorologie Dynamique, IPSL, CNRS, ENS, Université PSL, Sorbonne Université, École Polytechnique, Paris 75005, France

***Correspondence:** kepiyu@gmail.com (P.K.), philippe.ciais@cea.fr (P.C.)

**Maps of monthly terrestrial CO_2_ fluxes from emulators of Dynamic Global Vegetation Models**

We applied a deep-learning emulator framework identical to that used for oceanic fluxes[^[22]^](https://www.zotero.org/google-docs/?1C9EKe) to estimate monthly terrestrial carbon fluxes. Specifically, we utilized monthly terrestrial CO_2_ flux data from simulation 2 of 20 Dynamic Global Vegetation Models (DGVMs) included in the Global Carbon Budget 2025 [^[15]^](https://www.zotero.org/google-docs/?bcM1aa), covering the period up to December 2024 at a standardized spatial resolution of 0.5° × 0.5°. Predictor variables comprised atmospheric CO_2_ mole fraction, vegetation maps, lagged carbon fluxes from the previous 12 months and eight meteorological variables from the ERA5 [^[41]^](https://www.zotero.org/google-docs/?3An4a4): surface pressure, surface downward short-wave radiation, surface downward long-wave radiation, 2-meter temperature, total precipitation, 10-meter wind components, and specific humidity. All predictors were bilinearly interpolated to the uniform monthly spatial resolution. For the H1-2025 estimates used in this study, the final emulators were trained using monthly data through December 2024 and then applied to predict January–June 2025 fluxes. To make the emulator validation more self-contained, we summarize here the held-out validation results obtained with the same emulator framework and training strategy. In that validation experiment, 2022–2023 was withheld as an independent test period, while the emulators were trained and validated using data up to 2021. For monthly deseasonalized CO_2_ flux anomalies, individual emulators achieved R^2^ values of 0.39–0.94 and RMSE values of 0.03–0.19 GtC month^−1^ during the 2022–2023 test period. The DGVMs mean emulator performed better than most individual emulators, with R^2^ = 0.94 and RMSE = 0.03 GtC month^−1^. For monthly total terrestrial CO2 fluxes, individual emulator R^2^ values during the test period ranged from 0.91 to 1.00, with RMSE values of 0.03–0.21 GtC month^−1^; the DGVMs mean emulator achieved R^2^ = 1.00 and RMSE = 0.03 GtC month^−1^. These validation results show that the emulator framework reproduces the temporal variability of the parent DGVMs at aggregated scales, while also indicating larger model-dependent uncertainty for flux anomalies than for total seasonal fluxes.

The DGVM emulator ensemble also provides an independent comparison for the land-flux interpretation in this study (Supplementary Fig. 7). The emulators, trained on the land-model ensemble of the Global Carbon Budget 2025, reproduce the broad timing of the land-flux anomaly across 2023–2025: a shift to anomalous carbon losses in late 2024, followed by moderation but not full recovery in early 2025. This confirms that the temporal structure of the anomaly is not unique to the three low-latency DGVMs used in the main bottom-up budget. In particular, both the three-model emulator subset and the full 20-model emulator ensemble capture the prolonged period of negative or near-neutral anomalies extending from late 2024 into the first half of 2025. At the same time, the emulators generally produce a smaller amplitude of anomaly than the low-latency DGVMs, especially during the most negative months in late 2024. This difference is scientifically useful rather than problematic. It suggests that the sign and timing of the 2024–25 sink deficit are robust across model families, whereas the exact magnitude remains sensitive to model structure and forcing. That interpretation is also consistent with the comparison between bottom-up and top-down budgets in Section 2.1: the land sink clearly recovered relative to 2023–24, but the magnitude of its remaining deficit depends on the observing system and model framework used to estimate it. The emulator results therefore strengthen the central conclusion of this study while tempering the amplitude of the most negative bottom-up anomalies.

**Supplementary Fig. 1 Bivariate plots showing co-variations between net land flux anomalies from inversions and total water storage anomalies from the GRACE satellites in January-June 2025 (a), July-December 2024 (b) and January-June 2024 (c).** Green areas show wetter anomalies coincident with more CO_2_ uptake, and magenta areas show dryer anomalies that are coincident with reduced CO_2_ uptake.

**Supplementary Fig. 2 Monthly contributions of four latitude bands to global temperature anomalies and corresponding net land CO_2_ flux anomalies from 2015 to June 2025.** The upper panel shows the contributions of 60°N–90°N, 20°N–60°N, 20°S–20°N, and 60°S–20°S to the global mean temperature anomaly. The middle and lower panels show monthly net land CO_2_ flux anomalies from the DGVM ensemble and the atmospheric inversion ensemble, respectively, partitioned into the same latitude bands. Land-flux anomalies are relative to the 2015–2022 monthly mean. The shaded interval marks the July 2024–June 2025 anomaly period. More negative land-flux anomalies indicate reduced net land CO_2_ uptake or anomalous carbon release.

**Supplementary Fig. 3 Spatial patterns of climate-driven contributions to the July 2024–June 2025 net land CO_2_ flux anomaly.** Panels **(a)** and **(b)** show the temperature-driven component for the DGVM and atmospheric inversion ensembles, respectively; panels **(c)** and **(d)** show the corresponding terrestrial water storage (TWS)-driven component; and panels **(e)** and **(f)** show the radiation-driven component. These maps were derived from the same pixel-wise linear climate model used in Fig. 5, in which net land CO_2_ flux anomalies are decomposed into contributions from temperature, TWS and radiation. Each panel represents the mean contribution of the respective driver to the July 2024–June 2025 anomaly. Negative values indicate reduced net land CO_2_ uptake, or anomalous carbon release, relative to the reference period, whereas positive values indicate enhanced net land CO_2_ uptake.

**Supplementary Fig. 4 Bivariate plots showing co-variations between net land flux anomalies from DGVMs and total water storage anomalies from the GRACE satellites in January-June 2025 (a), July-December 2024 (b) and January-June 2024 (c).** Green areas show wetter anomalies coincident with more CO_2_ uptake, and magenta areas show dryer anomalies that are coincident with reduced CO_2_ uptake.

**Supplementary Fig. 5 Agreement between observed and climate-model-reconstructed distributions of non-overlapping 3-month net land CO_2_ flux anomalies.** Panels compare reconstructed and observed quantiles for global land, the tropics (23.5°S–23.5°N), and the Northern Hemisphere extratropics (>23.5°N), shown separately for the DGVM ensemble (top row) and the atmospheric inversion ensemble (bottom row). The reconstructed anomalies were derived from the same pixel-wise linear climate model used in the main text, in which net land CO_2_ flux anomalies are decomposed into contributions from temperature, terrestrial water storage (TWS), and radiation. The dashed line indicates the 1:1 relationship. Reported slopes and R^2^ values are obtained from linear regression between observed and reconstructed quantiles and summarize the similarity of the two anomaly distributions in each region.

**Supplementary Fig. 6 Spatial patterns of observed, climate-model-reconstructed and residual net land CO_2_ flux anomalies during July 2024–June 2025.** Panels **(a)** and **(d)** show the observed mean net land CO_2_ flux anomaly for the DGVM and atmospheric inversion ensembles, respectively. Panels **(b)** and **(e)** show the corresponding anomaly reconstructed from the same pixel-wise linear climate model used in Fig. 5, in which net land CO_2_ flux anomalies are decomposed into contributions from temperature, terrestrial water storage (TWS) and radiation. Panels **(c)** and **(f)** show the residual anomaly, calculated as observed minus reconstructed. All fields represent the mean anomaly over July 2024–June 2025 relative to the 2015–2022 reference period. Negative values indicate reduced net land CO_2_ uptake, or anomalous carbon release, relative to the reference period, whereas positive values indicate enhanced net land CO_2_ uptake. Negative residuals indicate regions where the observed anomaly was more source-like, or less sink-like, than predicted by the climate model.

**Supplementary Fig. 7 Comparison of monthly tropical CO_2_ flux anomalies from January 2023 to June 2025 between ML emulators of DGVMs and the three DGVMs used in this study.** **(a)** Comparison of ML emulators (yellow) and original simulations (green) for the same three DGVMs used in this study (ORCHIDEE, LPJ-EOSIM and JULES). The emulators are trained using runs of each DGVM until 2024 provided for the global carbon budget (GCB2025) [^[15]^](https://www.zotero.org/google-docs/?cVjkLL). The ORCHIDEE and LPJ-wsl models used in GCB2025 are different from the ORCHIDEE-MICT and LPJ-EOSIM versions used in this study. **(b)** Comparison of the three low latency DGVMs (green) used in this study with the emulators (yellow) of 20 DGVMs from GCB2025 with monthly outputs.

**Supplementary Fig. 8. Individual DGVM and inversion estimates of regional net land CO_2_ flux anomalies.** Anomalies are shown for July 2024–June 2025, July–December 2024, January–June 2025, JFM 2025, and AMJ 2025. Green circles denote individual DGVM members and blue triangles denote individual inversion members. Diamonds and error bars show the ensemble mean ±1 s.d. Positive values indicate stronger-than-normal CO_2_ uptake, whereas negative values indicate reduced uptake or anomalous release relative to the 2015–2022 reference period. The diagnostics are used to assess whether DGVM–inversion differences are driven by individual ensemble outliers or by systematic differences between the two approaches.

**Supplementary Fig. 9. Spatial difference between DGVM and inversion net land CO_2_ flux anomalies in early 2025.** Maps show the DGVM ensemble-mean anomaly minus the inversion ensemble-mean anomaly for JFM 2025 and AMJ 2025. Positive values indicate regions where DGVMs are more sink-like than inversions, whereas negative values indicate regions where DGVMs are more source-like than inversions. Anomalies are calculated relative to the 2015–2022 reference period and expressed in gC m^-2^ month^-1^. The maps are intended as diagnostic difference maps to identify regions of disagreement between bottom-up and top-down constraints.

**Supplementary Fig. 10. Ocean CO_2_ flux anomalies during the 2024–2025 ENSO transition.** (a) Global July–June ocean CO_2_ sink from the low latency ocean product ensemble. Grey shading denotes the product range, the black line the ensemble mean, the dashed line the 2015–2022 mean, and the red point July 2024–June 2025. (b) Quarterly tropical Pacific ocean CO_2_ flux anomalies integrated over 20°S–20°N, 160°E–80°W. (c) JFM 2025 ocean CO_2_ flux anomalies relative to the 2015–2022 seasonal mean. (d) AMJ 2025 ocean CO_2_ flux anomalies relative to the 2015–2022 seasonal mean. Dashed boxes in (c,d) mark the tropical Pacific region used in panel (b). Positive values indicate stronger uptake; negative values indicate reduced uptake or enhanced outgassing.

**Supplementary Table 1. Sensitivity of the bottom-up budget residual to atmospheric CO_2_ growth rate estimates for July 2024–June 2025.**

| **Atmospheric growth-rate product** | **CO_2_ growth rate (ppm yr^-1^)** | **Atmospheric accumulation (GtC yr^-1^)** | **Bottom-up residual (GtC yr^1^)** | **Implied land sink to close budget (GtC yr^1^)** |
| --- | --- | --- | --- | --- |
| NOAA MBL | 2.62 ± 0.08 | 5.55 ± 0.17 | 1.05 ± 0.70 | 1.61 ± 0.65 |
| OCO-2 | 2.68 ± 0.10 | 5.69 ± 0.21 | 0.92 ± 0.71 | 1.47 ± 0.67 |
| GRESO | 2.83 ± 0.10 | 6.01 ± 0.21 | 0.60 ± 0.71 | 1.15 ± 0.67 |

Note: Atmospheric accumulation was calculated using 1 ppm CO_2_ = 2.124 GtC. The residual is calculated as fossil emissions minus atmospheric accumulation, ocean sink, and DGVM land sink. Positive values indicate an additional sink required to close the bottom-up budget. Central residual values were calculated using fossil emissions = 10.35 GtC yr^-1^, the mean ocean sink = 3.19 GtC yr^-1^, and the mean DGVM land sink = 0.55 GtC yr^-1^. Uncertainties are 1-sigma values from the Monte Carlo propagation described in Methods. Fossil emissions were held fixed at the central low-latency estimate because the 10.33–10.37 GtC yr^-1^ range reported in the main text represents the spread between two near-real-time estimates rather than a formal 1-sigma uncertainty. The Monte Carlo 95% confidence intervals of the residuals include zero for all three atmospheric growth rate products; therefore, none of the residuals is significantly different from zero at the 95% confidence level.
